# Establishing an experimental model for canine atopic dermatitis through epicutaneous application of *Dermatophagoides farinae*

**DOI:** 10.3389/fvets.2022.1015915

**Published:** 2022-10-20

**Authors:** Sang-Won Kim, Jung-Hyun Kim

**Affiliations:** Department of Veterinary Internal Medicine, College of Veterinary Medicine, Konkuk University, Seoul, South Korea

**Keywords:** canine atopic dermatitis (CAD), experimental model, house dust mite (HDM), *Dermatophagoides farinae*, epicutaneous

## Abstract

There is no established protocol for the development of an experimental canine atopic dermatitis model in laboratory beagles. This study aimed to establish an experimental model that mimics spontaneous canine atopic dermatitis (CAD) clinically, immunologically, and microbiologically, by repeated epicutaneous applications of mite antigens and to describe the entire process including sensitization and provocation in detail for reproducibility. Six intact male laboratory beagle dogs aged 14 months were included in this study. During the sensitization and provocation phase, the house dust mite (HDM) paste consisted of *Dermatophagoides farinae* (*Der f* ) and mineral oil, which was applied focally to the 10 × 10 cm area of the right groin as evenly as possible. Further, 120 mg of *Der f* was applied to each dog twice a week for 12 weeks during the sensitization phase and 25 mg and 120 mg was applied to each dog for the first 2 weeks and subsequent 2 weeks, respectively, during the provocation phase. Thereafter, the applied area was covered with a dressing. Skin lesions including erythema, hyperpigmentation, excoriation, and lichenification were induced and exacerbated gradually through the experimental time course in all six dogs. The canine atopic dermatitis extent and severity index (CADESI) score and transepidermal water loss (TEWL) significantly increased after sensitization and provocation. IL-13 and IL-31 levels increased significantly after provocation as a result of the activation of the T helper-2 (Th2) response. On the contrary, the IL-10 levels decreased significantly after sensitization, which suggested a suppression of Tregs activity. After the completion of provocation, skin microbiome analysis showed that Firmicutes was the most abundant phylum, which indicated bacterial dysbiosis. This study demonstrated that epicutaneous application of HDM in beagle dogs resulted in the elevation of serum HDM-specific IgE levels and clinical atopic scores, a high TEWL, and microbiome dysbiosis resembling spontaneous CAD. These results suggest that this tailored protocol of epicutaneous exposure to *Der f* may provide support for the development of the experimental CAD model in laboratory beagles.

## Introduction

Canine atopic dermatitis (CAD) is a genetically predisposed inflammatory and pruritic skin disease with characteristic clinical features; its prevalence is approximately 10% in the canine population (1, 2). It is most commonly associated with hypersensitivity reactions to environmental allergens, especially house dust mites (HDM) including *Dermatophagoides farinae (Der f)* and *Dermatophagoides pteronyssinus (Der P)* (1, 3).

Similarly, the prevalence of atopic dermatitis is over 10% in human beings (2). There are numerous similarities between CAD and its human counterpart, suggesting that establishing a CAD model would be beneficial for researching both canine and human forms of the disease (4). For instance, the model would enable in-depth comprehension of the pathogenesis of atopic dermatitis and rapid screening of specific treatments prior to long clinical trials (4).

Researchers have worked on CAD models and attempted various protocols to induce the disease for decades, with one study identifying that simple repetitive epicutaneous exposure to the selected allergen can sensitize the dog (5, 6). In their model, atopic dermatitis (AD) lesions were induced through simple epicutaneous exposure to HDM, and no skin disruption was needed to induce sensitization (6). However, they used Maltese-Beagle dogs developed in their laboratory; these are at a high allergy risk. Furthermore, they only identified the validity of the sensitization protocol; the whole process of model development including sensitization and provocation was not described.

Cytokines play a major role in the development, differentiation, and function of cells such as lymphocytes, eosinophils, mast cells, and dendritic cells, all of which participate in the pathogenesis of atopic dermatitis (7). The initial acute phase is induced by the T helper-2 (Th2) response, which then leads to chronic T helper-1 (Th1) response (7).

A damaged skin barrier increases the risk of allergic sensitization and contributes to a dysregulated immune response, which is characteristic of CAD (8, 9). A non-invasive method used to assess the skin barrier function is the measurement of transepidermal water loss (TEWL), and higher TEWL values have been reported in atopic dogs than in healthy dogs (9).

Normal skin microbiome is necessary for proper skin function that regulates the innate immune response and prevents pathogenic microorganisms from colonizing (10). The skin microbial communities of CAD dogs are significantly different from those of healthy dogs; specifically, CAD dogs show a lower microbial diversity than healthy dogs (10, 11). This imbalance in the skin microbiota is called bacterial dysbiosis, and most studies have primarily focused on *Staphylococcus pseudintermedius*, which is the main pathogenic species in CAD (12).

The goal of this study was to establish an experimental model that mimics spontaneous CAD clinically, immunologically, and microbiologically, through repeated epicutaneous applications of mite antigens. To ensure that the model mimics spontaneous CAD, several measurements and analysis were undertaken. We have also tried to describe the entire process including sensitization and provocation in detail. These details include periods, intervals, and accurate doses of HDM application during sensitization and provocation and post application procedures so that researchers can replicate the entire protocol.

## Materials and methods

### Study design

**Figure 1:**
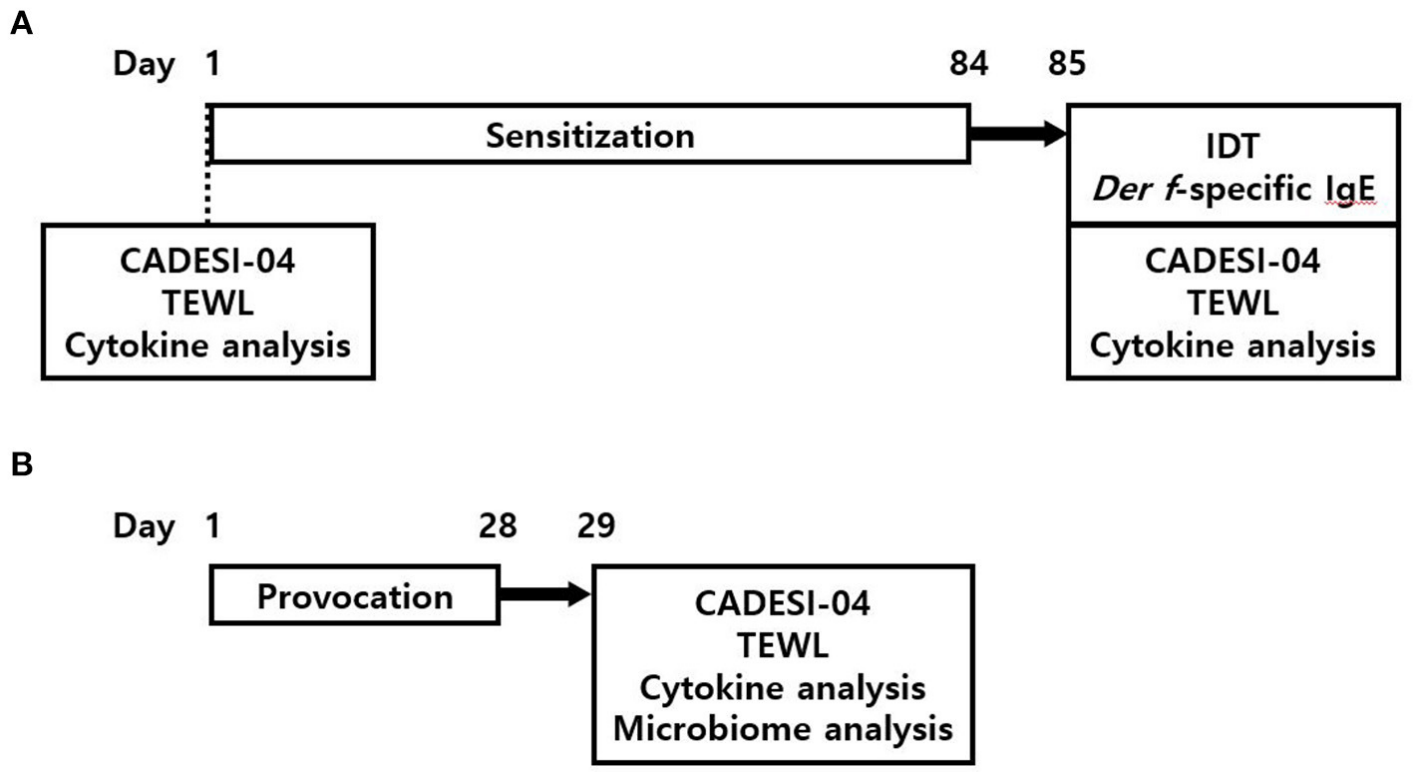
An outline of the study. **(A)** In phase 1, six dogs were sensitized with *Der f* for 12 weeks; then, the IDT and *Der-f* specific IgE assay were performed as a means of verification of sensitization. **(B)** Phase 2 began three days after completion of phase I. *Der f* challenges for 4 weeks were conducted as part of provocation. An exposure to *Der f* was repeated twice a week at three-day and four-day intervals alternately, during both phase 1 and 2. Clinical scoring including CADESI-04 and TEWL, and serum cytokine analysis was performed at pre-sensitization, after-sensitization, and after-provocation. The skin microbiome analysis was also conducted after-provocation. *Der f*, *Dermatophagoides farinae*; CADESI, canine atopic dermatitis extent and severity index; TEWL, transepidermal water loss.

This study consisted of two phases. In phase 1, we sensitized six beagle dogs with *Der f* for 12 weeks and verified the sensitization using an intradermal test and a *Der f*-specific IgE assay. In phase 2, we carried out mite challenges on the same site for 4 weeks. Before sensitization and at the end of phase 1 and 2, we performed clinical scoring using the canine atopic dermatitis extent and severity index (CADESI-04), measured the TEWL from the same site, and took a blood sample for circulating cytokine analysis. After the end of phase 2, we took a skin swab from the challenge site and analyzed the skin microbiome (Figure 1).

### Study subjects

Six laboratory beagle dogs were included in this study. All dogs were 14-month-old intact males, and their mean weight was 11.8 kg (range 10.8–12.7). Their sire and dam lines were all different and they were not genetically related to each other. Before the experiment, they were acclimated for 2 weeks, and there were no historical and clinical findings suggestive of dermatitis or any other allergic disease. During the entire period of the study, the 6 dogs were kept indoors in one room of the Konkuk Laboratory Animal Research Center, where the temperature and humidity were maintained at 22–24°C and 45–55%, respectively. Other dogs were not allowed to enter this room to limit their exposure to other allergens. Further, access to the dogs was restricted to people directly involved in this study. They were raised in individual runs made of stainless steel and the runs were cleaned using high pressure water once daily. Kennels were 1.7 cubic meters in size and large enough for dogs to move around freely. Items that could trap the mite bodies and cause consistent exposure to them such as bedding, clothes, and toys were all prohibited. They were fed conventional dry dog food based on daily energy requirements and could drink clean tap water voluntarily. To minimize disturbance to the skin condition, bathing was prohibited during the study.

### Materials

We made an HDM paste with *Der f* mite bodies (18M03, CITEQ biologics, Groningen, Netherlands) and mineral oil so that it was easy to apply on the skin. Before the suspension, we thawed the freeze-dried mite bodies for one hour. Thereafter, 6 g of mite bodies was suspended in 25 ml of mineral oil to achieve the concentration of 240 mg/ml. The paste was stored in a deep freezer at −80°C. Prior to every application, we thawed and vortexed the HDM paste for even consistency, and aliquots were made from this paste. A precise balance (FX-300i, A&D Company, Tokyo, Japan) was used when making an HDM paste or when collecting the aliquots.

### Study phase 1: Sensitization

#### Protocol for sensitization

**Figure 2:**
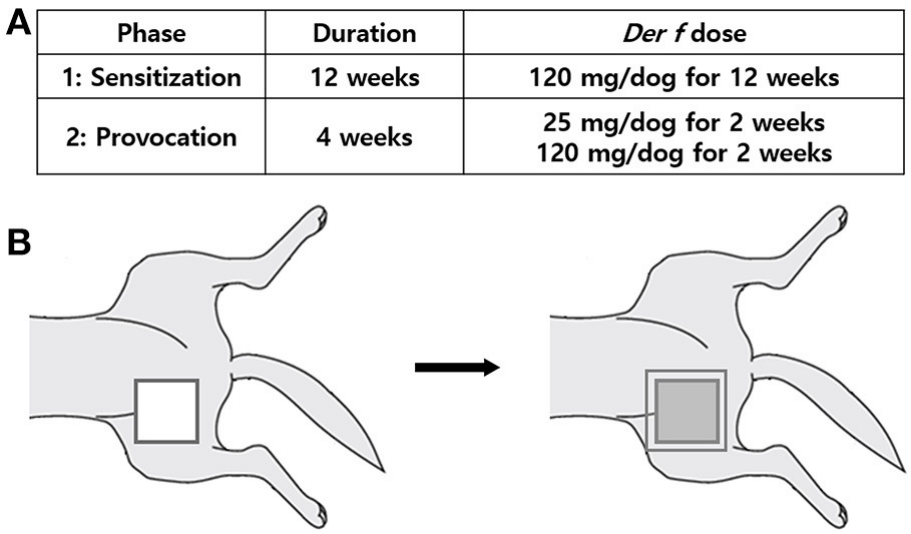
HDM paste application protocol. **(A)** Duration and applied *Der f* dose in study phase 1 (Sensitization) and phase 2 (Provocation). **(B)** Application procedure. Clip the hair of the right groin, apply the HDM paste directly to 100 cm^2^ area of the right groin skin as evenly as possible, then secure the application site with transparent bio-occlusive dressing, and place the plastic e-collars on the dogs. *Der f*, *Dermatophagoides farinae*.

We decided to apply the HDM paste focally on the right groin. Before every application, hair on the right groin was clipped. Thereafter, 0.5 ml of HDM paste was used per dog; therefore, 120 mg of Der f was applied to each dog. The HDM paste was directly applied to 10 × 10 cm area of the intact skin of the right groin as evenly as possible. Then, the area was secured with a transparent bio-occlusive dressing (Tegaderm™, 3M, St. Paul, MN, USA) and plastic e-collars were placed on the dogs. Dressing and e-collars were removed on the very next day. Each dog had a total of 12 week-exposure to the patch at the same site that was repeated twice a week at three-day and four-day intervals alternately (Figure 2).

#### Verification of sensitization

After the completion of the sensitization protocol, *Der f*-specific IgE assay and an intradermal test were performed to verify the sensitization. Blood samples (1 ml) were obtained from all the dogs and serum samples were sent to Heska AG (Fribourg, Switzerland) for ALLERCEPT^®^ *Der f*-specific IgE assay without any information about the study. According to the manufacturer's criterion, 11–25 Heska Epsilon Receptor Binding Units (HERBU) are considered “+,” 26–50 HERBU are considered “++,” and >50 are considered “+++.” The intradermal test was carried out on dorsum skin. The diluted *Der f* extract (HollisterStier, Spokane, WA, USA) was prepared at a concentration of 1,000 AU/ml. Thereafter, histamine solution was injected as a positive control, normal saline as negative control, and mite extracts were injected intradermally at 0.1 ml each with a 27-gauge needle. The diameter of the erythematous wheals was measured 15 min after the injection. Skin reactions were considered positive when *Der f* yielded a wheal larger than half the diameter of the positive control.

### Study phase 2: Provocation

#### Protocol for provocation

Provocation was conducted using the same HDM paste and at the same skin region, but it differed from sensitization in the duration and application dose of *Der f*. Application was repeated twice a week for 4 weeks at 25 mg doses of *Der f* for the first 2 weeks and 120 mg for the following 2 weeks. Intervals between each application and application and post application procedures were identical to the sensitization protocol (Figure 2).

### Evaluation of skin lesions

The severity of skin lesions was scored based on CADESI-04, the validated scale to score skin lesions of CAD. Erythema, lichenification, and excoriation lesions of the right groin were scored from 0 to 3 for a maximum score of 9 points. It was performed by the same veterinarian who was blinded to the study details.

### Evaluation of skin barrier function

The barrier quality of the stratum corneum was evaluated based on TEWL. It was measured in the right groin region using a VapoMeter^®^ (Delfin Technologies, Kuopio, Finland), and every measurement was performed in the same room where the dogs lived. Five successive values were obtained, and the mean values were calculated.

### Serum cytokine analysis

Blood samples (5 ml) were obtained from the jugular vein, and inflammatory cytokines were measured in serum samples. Commercially available ELISA kits, which are labeled to detect canine IFN-gamma (DY781B, R&D Systems, Minneapolis, MN, USA), IL-2 (DY1815, R&D Systems, Minneapolis, MN, USA), IL-4 (DY754, R&D Systems, Minneapolis, MN, USA), IL-10 (DY735, R&D Systems, Minneapolis, MN, USA), IL-12 (DY1969, R&D Systems, Minneapolis, MN, USA), IL-13 (SEA060Ca, Cloud-Clone Corp., Katy, TX, USA), IL-31 (ECI0041, ABclonal, Woburn, MA, USA), and TGF-beta (DB100B, R&D Systems, Minneapolis, MN, USA), were used.

### Microbiome sample collection

Skin swab samples were collected from the right groin after the completion of the provocation protocol, using eSwab™ (COPAN Diagnostics, Murrieta, CA, USA), which is a sterile culture swab applicator. The swab samples were stored at −80 °C and further bacterial DNA extraction and 16S rRNA sequencing were performed.

### DNA extraction, PCR amplification, and sequencing

MiSeq (Illumina, San Diego, CA, USA), an integrated next generation sequencing instrument, was used. Total DNA was extracted in accordance with the manufacturer's instruction. PCR amplification of the extracted DNA was performed using fusion primers targeting the V3 to V4 regions of the 16S rRNA gene. For bacterial amplification, fusion primers 341F and 805R were used (Table 1).

**Table 1:** Primer sequences for PCR targeting the V3–V4 region of bacteria.

**Primer**	**Sequence**
341F	5'-AATGATACGGCGACCACCGAGATCTACAC-XXXXXXXX-TCGTCGGCAGCGTC-AGATGTGTATAAGAGACAG-*CCTACGGGNGGCWGCAG*-3'
805R	5'- CAAGCAGAAGACGGCATACGAGAT-XXXXXXXX-GTCTCGTGGGCTCGG-AGATGTGTATAAGAGACAG-*GACTACHVGGGTATCTAATCC*-3'

Italic sequences indicate the target region primer.

The amplifications were performed under the following conditions: initial denaturation at 95°C for 3 min, followed by 25 cycles of denaturation at 95°C for 30 s, primer annealing at 55°C for 30 s, extension at 72°C for 30 s, and final elongation at 72°C for 5 min.

The amplified products were purified with CleanPCR (CleanNA, PH Waddinxveen, the Netherlands) which pool the equal concentrations of purified products and remove the short fragments (non-target products). The quality and product size were assessed using Bioanalyzer 2,100 (Agilent, Palo Alto, CA, USA). Mixed amplicons were pooled, and sequencing was carried out at CJ Bioscience, Inc. (Seoul, Korea) with MiSeq.

### Data analysis

Processing raw reads began with a quality check and filtering of low-quality reads (<Q25) using Trimmomatic ver. 0.32. After QC, paired-end sequence data were merged together with default parameters using the fastq_mergepairs command of VSEARCH version 2.13.4. Then, primers were trimmed using the alignment algorithm of Myers & Miller at a similarity cut-off of 0.8. The nhmmer in HMMER software package ver. 3.2.1 detected non-specific amplicons that did not encode 16S rRNA. Unique reads were extracted, and redundant reads were clustered with the unique reads using the derep_fulllength command of VSEARCH. The EzBioCloud 16S rRNA database was used for taxonomic assignment using the usearch_global command of VSEARCH followed by more precise pairwise alignment. The reference-based chimeric detection using the UCHIME algorithm and the non-chimeric 16S rRNA database from EzBioCloud filtered chimeric reads with <97% similarity. After chimeric filtering, reads that were not identified to any species level in the EzBioCloud database were compiled and the cluster_fast command was used to carry out de-novo clustering to generate additional operational taxonomic units (OTUs). Finally, OTUs with single reads were omitted from further analysis. The alpha diversity indices, Chao1 and Shannon, were estimated by inhouse programs of CJ Bioscience, Inc (Seoul, South Korea). All analytics mentioned above were performed in EzBioCloud 16S-based MTP, which is a CJ Bioscience's bioinformatics cloud platform.

### Statistical analysis

Prism 8 software (Graphpad Software, San Diego, CA) was used for statistical analysis. To analyze the effect of epicutaneous application of HDM on CADESI-04, TEWL, and inflammatory cytokines, non-parametric Friedman's tests with Dunn's *post-hoc* tests were used. All values are expressed as the mean ± standard error.

## Results

### Verification of sensitization

**Figure 3:**
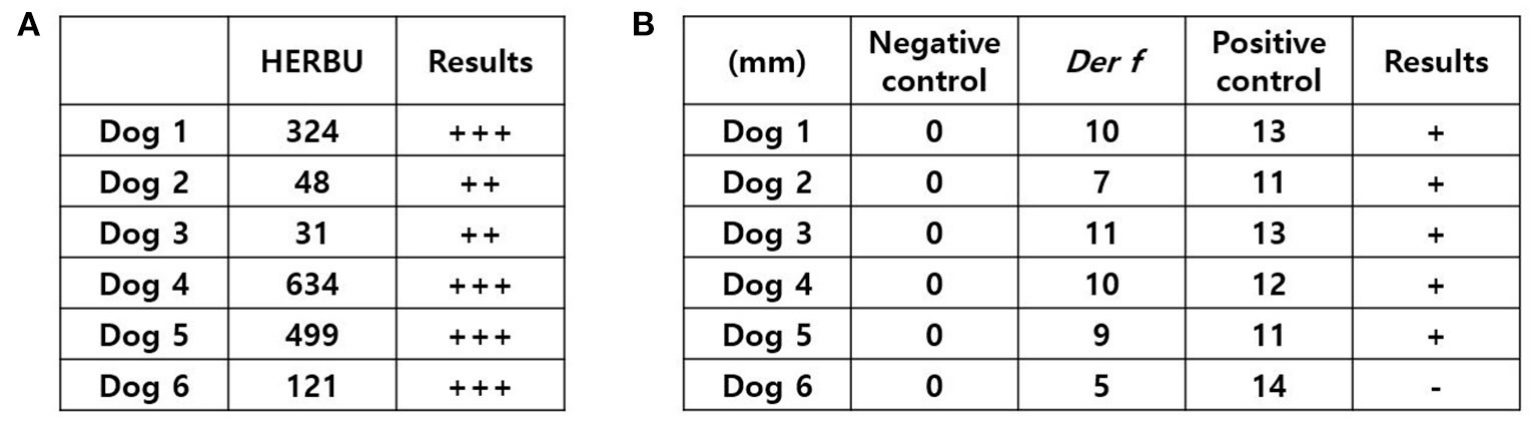
Verification of *Der f* sensitization. **(A)** Results of the *Der f*-specific IgE assay. **(B)** Results of the intradermal test. Dog 6 showed a wheal at the *Der f* injection site, which was not larger than half of that of a wheal at the histamine injection site; hence, it was evaluated as negative. HERBU, Heska Epsilon Receptor Binding Units; *Der f*, *Dermatophagoides farinae*.

In *Der f*-specific IgE assay, all dogs showed positive results, which were greater than or equal to two positives (Figure 3A). However, in the intradermal test (IDT), five dogs were evaluated as positive, but one dog was evaluated as negative. Dog 6, who had a negative IDT result, showed a wheal at the *Der f* injection site but its diameter was not larger than half the diameter of the wheal at the histamine injection site (Figure 3B).

### Evaluation of skin lesions

**Figure 4:**
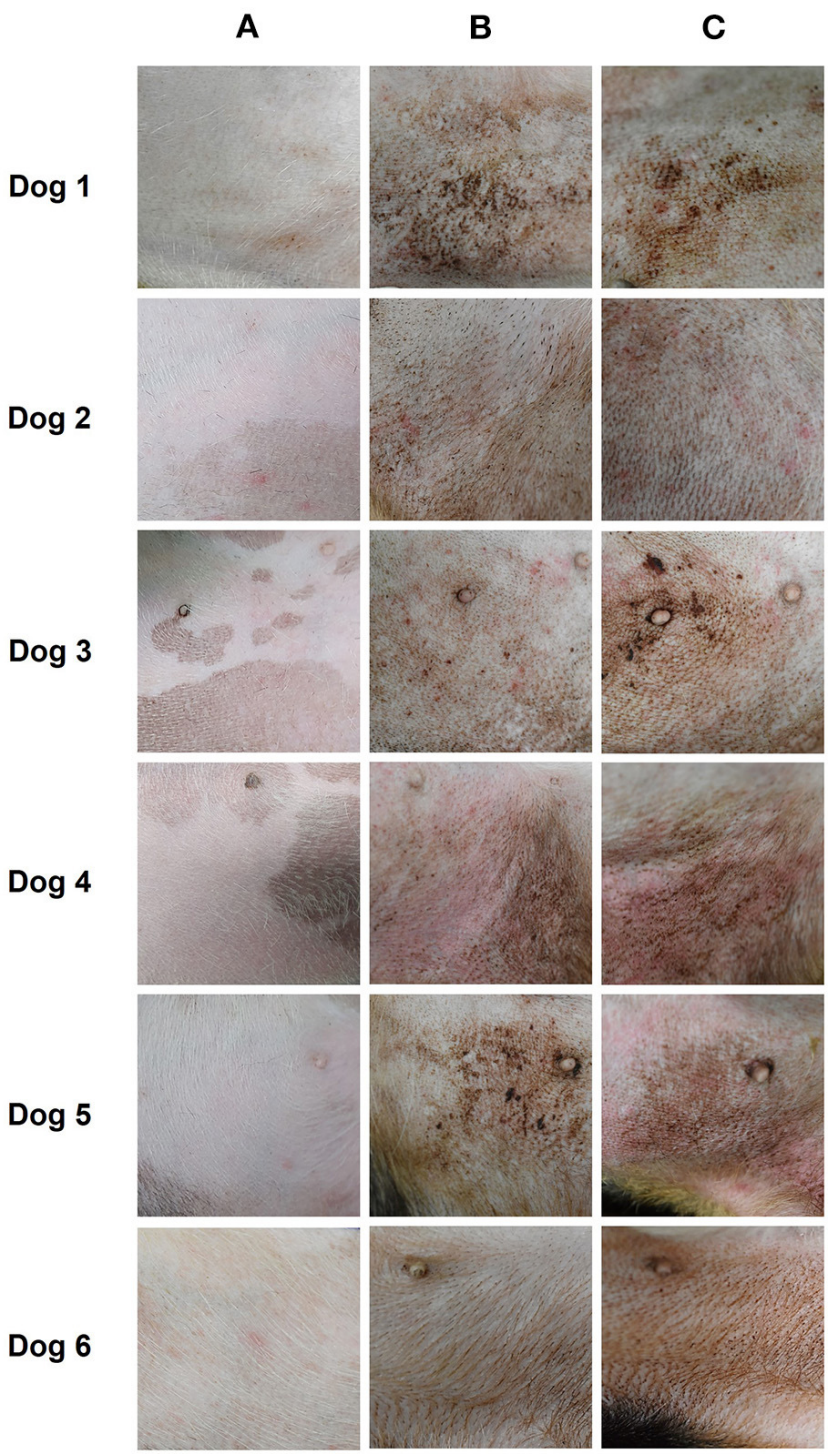
Gross skin lesions of the right groin area in six dogs. Images show the time course of development of the skin lesions at **(A)** pre-sensitization, **(B)** post-sensitization, and **(C)** post-provocation.

**Figure 5:**
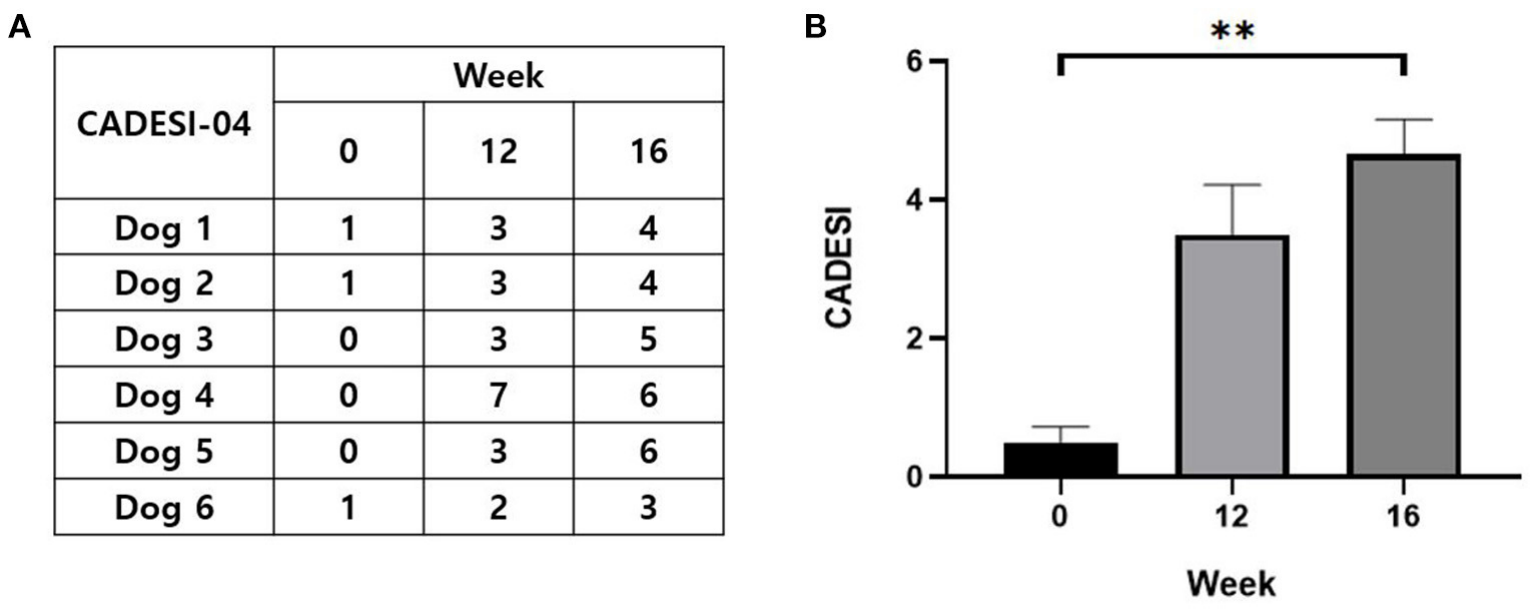
Evaluation of the skin lesions. **(A)** CADESI-04 score of each subject at pre-sensitization, post-sensitization, and post-provocation. **(B)** Change in the CADESI-04 with the course of time. Zero week, pre-sensitization; 12 week, post-sensitization; 16 week, post-provocation. CADESI, canine atopic dermatitis extent and severity index. All values are expressed as the mean ± standard error. **P < 0.01.

In the right groin area, skin lesions such as erythematous edema, excoriation, and lichenification developed in all the dogs after sensitization and provocation (Figure 4). The CADESI-04 score of each subject at pre-sensitization, post-sensitization, and post-provocation is presented in Figure 5A. CADESI-04 increased significantly from 0.5 ± 0.2 at pre-sensitization to 4.6 ± 0.4 at post-provocation (*P* < 0.01; Figure 5B).

### Evaluation of skin barrier function

**Figure 6:**
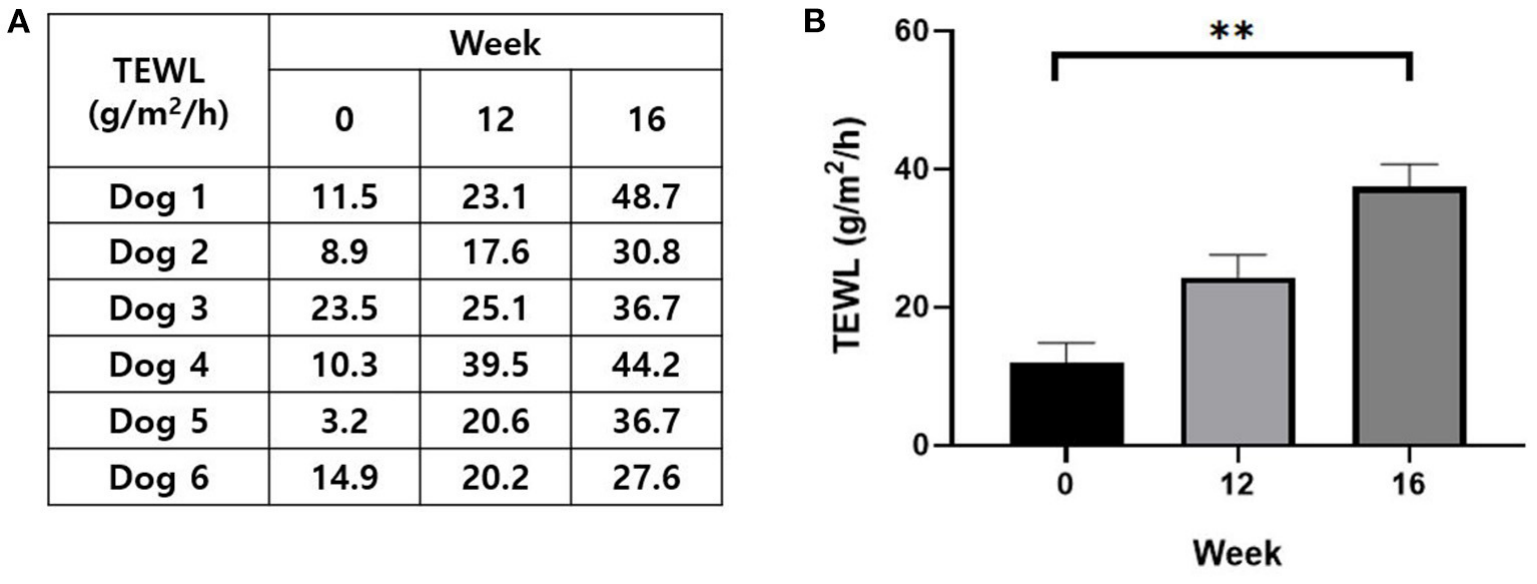
Evaluation of skin barrier function. **(A)** The TEWL value of each subject at pre-sensitization, post-sensitization, and post-provocation. **(B)** Change in the TEWL with the course of time. Zero week, pre-sensitization; 12 week, post-sensitization; 16 week, post-provocation. TEWL, transepidermal water loss. All values are expressed as the mean ± standard error. **P < 0.01.

The TEWL of each subject measured at pre-sensitization, post-sensitization, and post-provocation is presented in Figure 6A. TEWL increased significantly from 12.0 ± 2.7 g/m^2^/h at pre-sensitization to 37.4 ± 3.2 g/m^2^/h at post-provocation (*P* < 0.01; Figure 6B).

### Serum cytokine analysis

**Figure 7:**
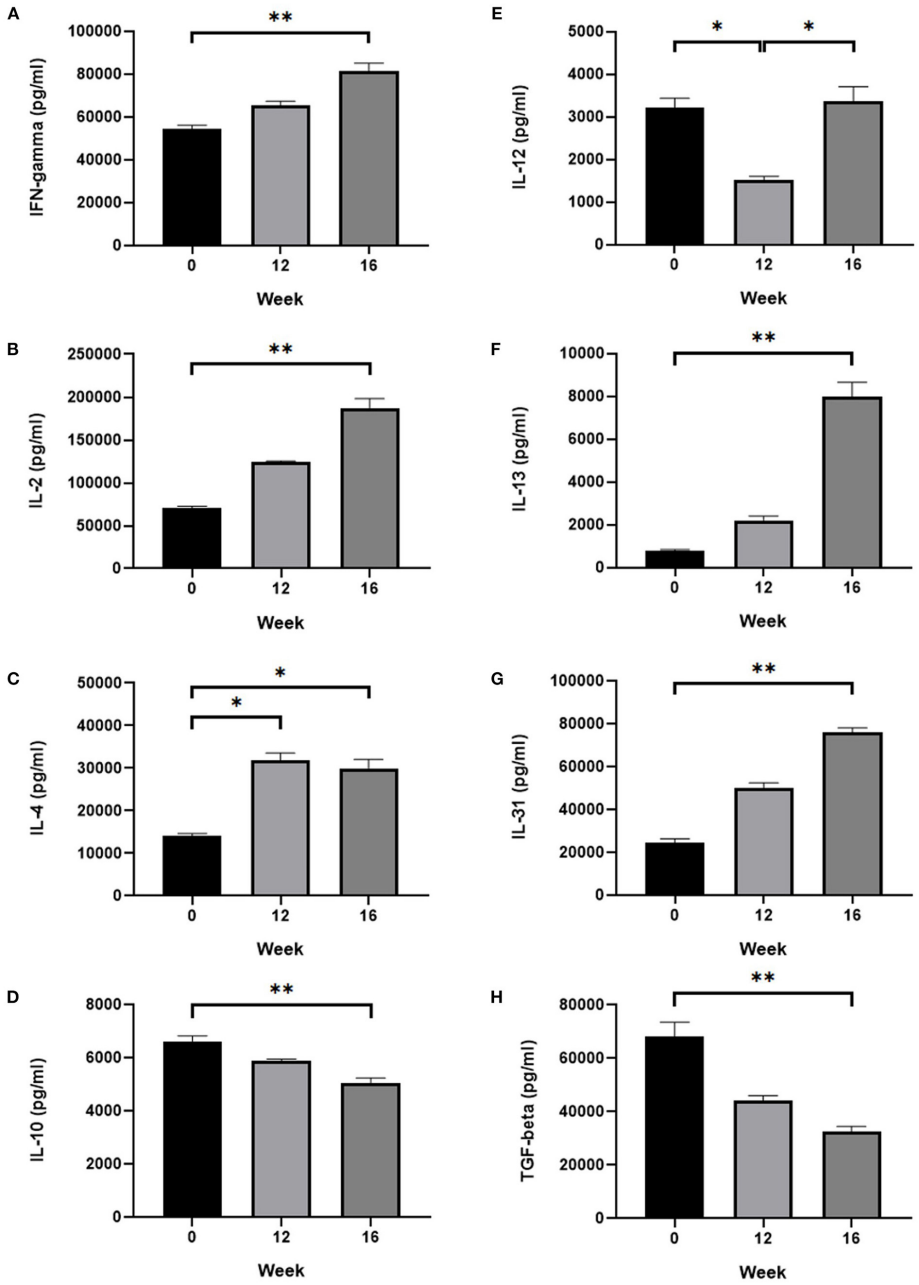
Serum cytokine analysis. Change in the serum level of **(A)** IFN-gamma, **(B)** IL-2, **(C)** IL-4, **(D)** IL-10, **(E)** IL-12, **(F)** IL-13, **(G)** IL-31, and **(H)** TGF-beta. IL-4, IL-13 and IL-31, which belong to the Th2 cytokine group, were increased significantly after provocation. IFN-gamma, IL-2, and IL-12, which belong to the Th1 cytokine group, did not show a common pattern over the time course. IL-10 and TGF-beta, which belong to the Treg cytokine group, were decreased significantly after provocation. Zero week, pre-sensitization; 12 week, post-sensitization; 16 week, post-provocation. All values are expressed as the mean ± standard error. *P < 0.05, **P < 0.01.

Significant increase in IFN-gamma was found from 54,240 ± 1,827 pg/ml at pre-sensitization to 81,318 ± 3,717 pg/ml at post-provocation (*P* < 0.01; Figure 7A). IL-2 increased significantly from 70,111 ± 2,501 pg/ml at pre-sensitization to 187,389 ± 10,904 pg/ml at post-provocation (*P* < 0.01; Figure 7B). IL-4 increased significantly from 14,022 ± 619.4 pg/ml at pre-sensitization to 31,783 ± 1,773 pg/ml at post-sensitization and 29,978 ± 2,082 pg/ml at post-provocation (*P* < 0.05, both; Figure 7C). IL-10 decreased significantly from 6,623 ± 199.7 pg/ml at pre-sensitization to 5,066 ± 168.8 pg/ml at post-provocation (*P* < 0.01; Figure 7D). IL-12 decreased significantly from 3,221 ± 213.1 pg/ml at pre-sensitization to 1,518 ± 82.8 pg/ml at post-sensitization and increased significantly from 1,518 ± 82.8 pg/ml at post-sensitization to 3,355 ± 350.2 pg/ml at post-provocation (*P* < 0.05, both; Figure 7E). IL-13 increased significantly from 791.2 ± 50.3 pg/ml at pre-sensitization to 7,976 ± 670.9 pg/ml at post-provocation (*P* < 0.01; Figure 7F). IL-31 increased significantly from 24,665 ± 1,645 pg/ml at pre-sensitization to 75,934 ± 2,102 pg/ml at post-provocation (*P* < 0.01; Figure 7G). TGF-beta decreased significantly from 68,199 ± 5,201 pg/ml at pre-sensitization to 32,467 ± 1,946 pg/ml at post-provocation (*P* < 0.01; Figure 7H).

### Skin microbiome analysis at the phylum level

**Figure 8:**
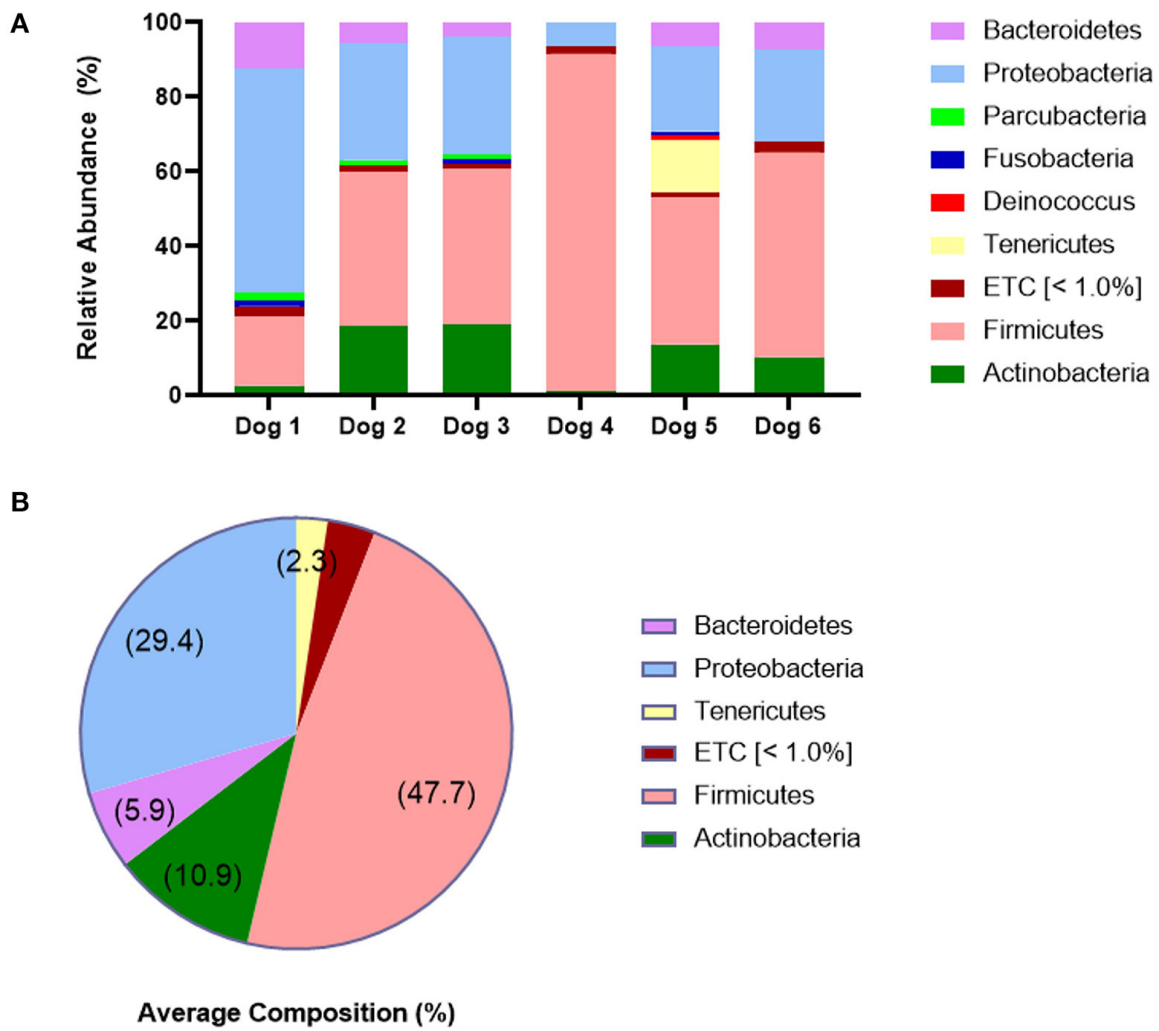
Skin microbiome analysis at the phylum level. **(A)** Individual composition of bacterial phyla and **(B)** average composition of bacterial phyla in six dogs at post-provocation. The most abundant phylum was Firmicutes, followed by Proteobacteria and Actinobacteria, which shows bacterial dysbiosis. The cut-off value for ETC taxa is 1.0%.

The individual and average composition of bacterial phyla in the six dogs post provocation is presented in Figure 8. The most abundant phylum in the six dogs was Firmicutes, followed by Proteobacteria, Actinobacteria, Bacteroidetes, and Tenericutes (47.7, 29.4, 10.9, 5.9, and 2.3%, respectively). Other taxa which account for less than 1.0% were merged into ETC taxon.

### Analysis of alpha diversity of the skin microbiome

**Figure 9:**
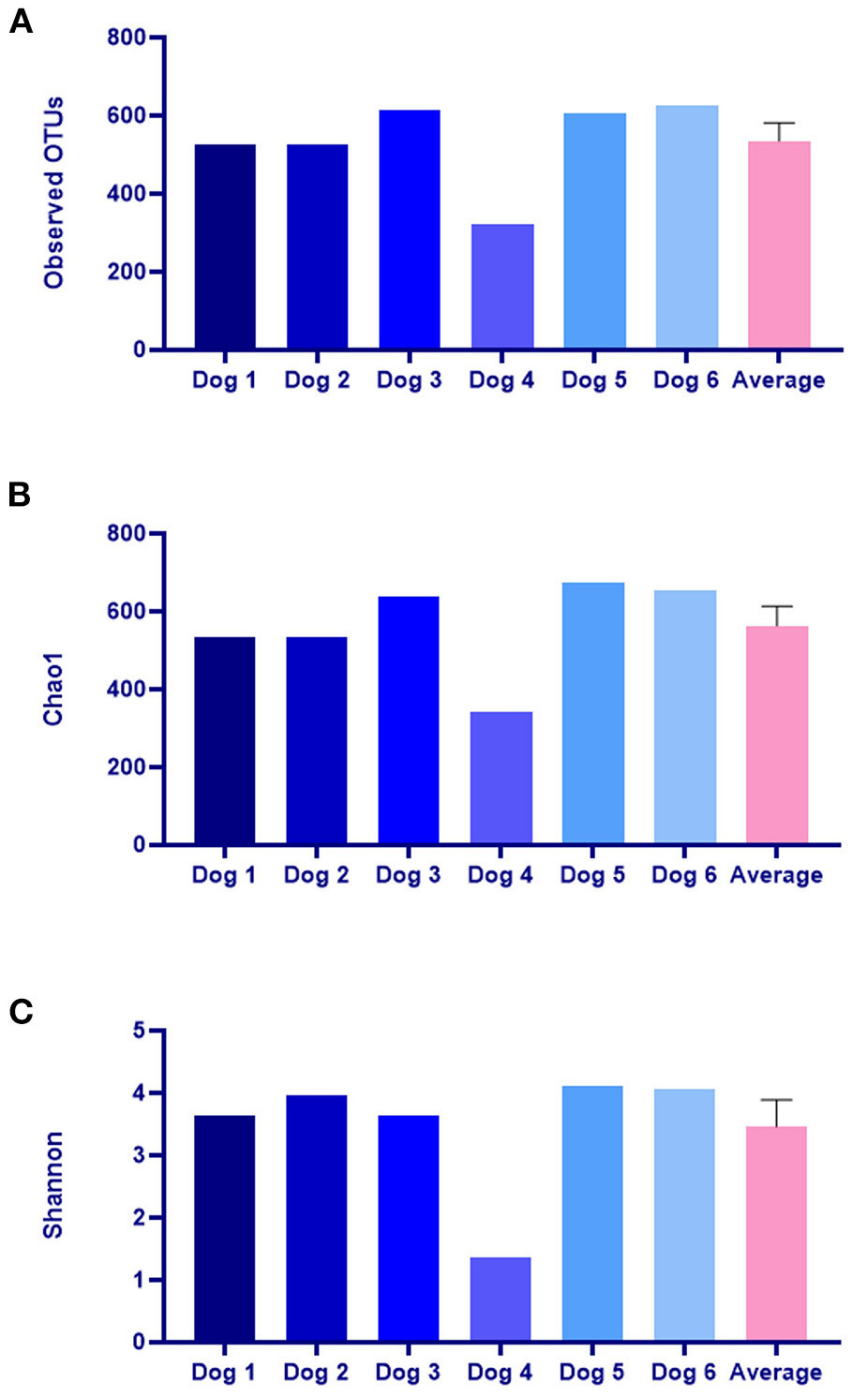
Analysis of alpha diversity of the skin microbiome including **(A)** observed OTUs, **(B)** Chao1, and **(C)** Shannon. OTUs, Operational Taxonomic Units. The average values are expressed as the mean ± standard error.

A total of 420,237 reads were obtained from skin swab samples of all the subjects, and alpha diversity indices were calculated after the reads were normalized (61,927 reads) in each sample (Table 2). Good's coverage values were over 99% in all subjects. The average values of the observed OTUs, Chao1, and Shannon for the six dogs were 535.1 ± 46.7, 562.6 ± 50.2, and 3.4 ± 0.4, respectively (Figure 9).

**Table 2:** Summary of the alpha diversity indices of the skin microbiome.

**ID**	**Total valid reads**	**Number of normalized reads**	**Observed OTUs**	**Chao1**	**Shannon**	**Good's coverage (%)**
1	61,927	61,927	524	532.84	3.63	99.92
2	62,883	61,927	524	535.76	3.95	99.92
3	69,584	61,927	613	637.29	3.64	99.86
4	63,194	61,927	320	343.61	1.37	99.92
5	97,316	61,927	605	672.78	4.12	99.84
6	65,333	61,927	625	653.88	4.06	99.88

OTUs, operational taxonomic units.

## Discussion

In this study, we established a protocol for experimental CAD model development, and examined the clinical and immunological effects of the epicutaneous application of *Der f* through the sensitization and provocation phase. During the entire period of the study, exposure to other allergens was strictly restricted by prohibiting the six dogs from exiting the room and limiting their access to other dogs and people. After the completion of provocation, skin microbial diversity and composition were analyzed to evaluate whether they were similar to those of spontaneous CAD dogs.

First, skin lesions including erythema, hyperpigmentation, excoriation, and lichenification were induced and exacerbated gradually through the experimental time course. These are the representative skin lesions in spontaneous CAD (13). In addition to the visible skin lesions, the CADESI-04 score increased significantly after sensitization and provocation. This validated scale used to evaluate the severity of CAD confirmed the deterioration of the skin lesions. Further, the increased excoriation and lichenification score indicated the induction of pruritus by epicutaneous application of *Der f* and its progression to a chronic state. TEWL, a non-invasive method used to assess the skin barrier function, also increased significantly after sensitization and provocation. It suggests the impairment of skin barrier after *Der f* application, specifically the stratum corneum, and it increases the risk of sensitization, resulting in vicious cycles of progressive skin damage (8).

Second, we analyzed the concentration of multiple pro-inflammatory and anti-inflammatory cytokines in peripheral blood to identify if this model mimics the spontaneous CAD immunologically. After the allergen penetrates through the defective skin barrier, it interacts with local immune cells and atopic dermatitis-related pro-inflammatory cytokines are released (14). Most studies regarding atopic dermatitis in dogs and humans reported that initial or acute response of Th2 represents the major cytokine response in the pathogenesis of atopic dermatitis (14–17). During the Th2 response, IL-4, IL-5, IL-13, and IL-31 predominate and these cytokines promote humoral immunity, including IgE production (16, 17). Among these cytokines, IL-31 plays a key role in pruritic skin diseases as a potent pruritogenic cytokine (18). Subsequently, the Th1 response follows the Th2 response and leads to the chronic phase of inflammation in human AD (14). Th1 type cytokines include IFN-gamma, IL-2, and IL-12 (16). In addition to the abovementioned Th types, regulatory T cells (Tregs) also exist. The main role of these cells is to suppress the activation of the allergen-specific Th2 response by secreting anti-inflammatory cytokines, including IL-10 and TGF-beta (19).

In the present study, Th2 cytokine levels increased significantly after provocation and IL-13 and IL-31 revealed an upward trend over time. The IL-31 level demonstrates the progressive deterioration of pruritus during the protocol. However, the IL-4 levels lacked a consistent trend over time. These results confirm the opinion presented in a previous study (7) that among the Th2 cytokines, IL-13 may play a more critical role in atopic response than IL-4. Moreover, IL-10 and TGF-beta levels decreased significantly after provocation and showed a downward trend over time. Likewise, these results correspond to the results of a previous study (7), in which CAD dogs showed lower plasma levels of IL-10 and TGF-beta than healthy dogs. It suggests that the activity of Tregs in beagle dogs was suppressed, resulting in the decrease of anti-inflammatory cytokine release and activation of allergen-specific T cell responses and further allergic processes. IFN-gamma and IL-2 level increased significantly after provocation, but IL-12 levels did not show a certain pattern over time. According to a previous study, a typical Th1 response is difficult to recognize; rather, a mixed Th1-Th2 response was observed in CAD unlike in human AD (7, 20). Therefore, the Th1 cytokines level identified in this study are also obscure and further research is required.

Lastly, we analyzed the skin microbiome post provocation and the most abundant phylum in the six dogs was Firmicutes, followed by Proteobacteria and Actinobacteria. According to a previous study (10), the most abundant phylum is Proteobacteria in both healthy and allergic dogs. On the other hand, Pierezan et al. (12) challenged the Maltese-beagle dogs with HDM epicutaneously for 3 consecutive days and observed that the most abundant phylum was Firmicutes in post-challenge samples, followed by Proteobacteria. They found significantly increased proportions of the phylum Firmicutes on challenge days 14, 21, and 28 compared to the pre-challenge sample, in which the most abundant phylum was Proteobacteria. These results demonstrate the bacterial dysbiosis after the challenge, and we also suspected that skin bacterial dysbiosis occurred through the sensitization and provocation procedure in all the six dogs in this study.

In CAD, flare state is associated with colonization or superficial infection by S. pseudintermedius or S. schleiferi (11). These species belong to the phylum Firmicutes and particularly, S. pseudintermedius is a main pathogenic species in CAD (21). Furthermore, previous studies identified that the abundance of S. pseudintermedius decreases after treatment of CAD (11, 22). Therefore, we suggested that the overgrowth of pathogenic species including S. pseudintermedius occurred and therefore the phylum Firmicutes became the most abundant in this study. Further studies are needed to verify the abundance of S. pseudintermedius in CAD models.

According to a previous study (11), alpha diversity indices including OTUs, Shannon, and Chao1 were lower in dogs with CAD than in healthy dogs. In addition, these diversity indices gradually increased with treatment and approached the mean value observed in the healthy dogs. These findings indicate that decreased microbial diversity in CAD dogs normalized with treatment. In our study, the alpha diversity indices in six dogs were much lower than those observed in dogs with CAD in the abovementioned study (11). Therefore, it can be assumed that skin microbiome dysbiosis was sufficiently induced. However, the differences in diversity indices before and after model production could not analyzed due to the lack of microbiome analysis before sensitization. Further studies are needed to verify the induction of bacterial dysbiosis in CAD models.

The first limitation of this study is the lack of histopathology and immunohistochemistry at the lesion site after CAD model development. Histopathology and immunohistochemistry are used to identify the numerous immune cell types which infiltrate the dermis, and to estimate the severity of inflammation induced based on the cell recruitment intensity (6, 23, 24). Instead, we analyzed the skin microbiome at the lesion site in this study because it has already been proven that the allergen challenge in sensitized dogs results in bacterial dysbiosis at the site of lesion (12). Therefore, the absence of microbiome analysis before sensitization is limitation of this study and needs to be accounted for in future studies. Second, negative IDT results were obtained in one dog after the completion of sensitization. However, the *Der f*-specific IgE titer result in this dog was “+++;” hence, induction of hypersensitivity to HDM was proven. According to the American College of Veterinary Dermatology (ACVD) task force on CAD, some dogs with naturally occurring CAD or who are experimentally sensitized to allergens could show the IgE-mediated late-phase reaction at 6–12 hours post injection in IDT (25). Since we did not examine the injection site at 6–12 hours post injection, it is possible that we missed the lesion and misinterpreted the result as negative. Lastly, this study did not include a control group to compare allergen specific IgE titer, cytokines concentration, or skin microbiome. Although the presence of a control group would have helped to ensure the model establishment, we deemed it to be non-essential since this was not a case-control study.

In earlier studies, some researchers mentioned that beagle dogs who were sensitized to HDM previously by the epicutaneous route were used in the experiment, but they did not describe the detailed sensitization protocol (4, 26–30). Furthermore, most epicutaneous exposures mentioned did not seem to be direct skin exposures but were environmental exposures, which is a mixture of epicutaneous, oral, and inhalation exposure (5). However, in two previous studies (31, 32), authors briefly described additional details about the sensitization procedure, but the results of verification including the clinical score, IDT, or HDM-specific IgE titers were not provided. For these reasons, there is no established CAD model development protocol in laboratory beagle dogs that researchers could follow.

The major strength of this study is that it describes the entire protocol for experimental CAD model development in detail, including sensitization and provocation. This study expands upon a previous study regarding epicutaneous sensitization conducted by Pucheu-Haston et al. (6), in which Maltese-Beagle dogs were used. These lines were developed in their laboratory and have a predisposition for spontaneous development of hypersensitivity to environmental allergens; hence, they are prone to food allergy and CAD (6). However, validity of the sensitization protocol was not proven in laboratory beagles, which is a widely used experimental breed. Lastly, we identified that the CAD models we made mimic the naturally occurring CAD dogs microbiologically. In a previous study (6), microbiological analysis was not conducted in the developed CAD models. Based on the results of this study, many researchers could produce the same experimental CAD model and use it for their research on CAD and AD in humans.

## Data availability statement

The datasets presented in this study can be found in online repositories. The name of the repository and accession numbers can be found below: NCBI; SRX17180029, SRX17180030, SRX17180033, SRX17180034, SRX17180035, SRX17180036.

## Ethics statement

The animal study was reviewed and approved by Konkuk University Institutional Animal Care and Use Committee.

## Author contributions

S-WK: conceptualization, formal analysis, investigation, resource, writing-original draft preparation, writing-review, and editing, and visualization. J-HK: conceptualization, writing-original draft preparation, writing-review and editing, supervision, and project administration. Both authors contributed to the article and approved the submitted version.

## Funding

This work was supported by the Korea Institute of Planning and Evaluation for Technology in Food, Agriculture and Forestry (IPET) through the Development of technology for immunomodulatory ability of useful exosomes derived from stem cells for companion animal Project, funded by Ministry of Agriculture, Food and Rural Affairs (MAFRA) (321013-01).

## Conflict of interest

The authors declare that the research was conducted in the absence of any commercial or financial relationships that could be construed as a potential conflict of interest.

## Publisher's note

All claims expressed in this article are solely those of the authors and do not necessarily represent those of their affiliated organizations, or those of the publisher, the editors and the reviewers. Any product that may be evaluated in this article, or claim that may be made by its manufacturer, is not guaranteed or endorsed by the publisher.
